# The Food Resources and Kitchen Skills intervention: Protocol of a randomized controlled trial

**DOI:** 10.1371/journal.pone.0314275

**Published:** 2025-02-06

**Authors:** Armando Peña, Emily Dawkins, Mariah Adams, Lyndsi R. Moser, Amy Carter, Rebecca L. Rivera, Deanna Reinoso, Wanzhu Tu, Richard J. Holden, Daniel O. Clark

**Affiliations:** 1 Department of Health & Wellness Design, School of Public Health-Bloomington, Indiana University, Bloomington, Indiana, United States of America; 2 Eskenazi Health, Indianapolis, Indiana, United States of America; 3 Department of Psychiatry, Indiana University School of Medicine, Indianapolis, Indiana, United States of America; 4 Department of Medicine, Indiana University School of Medicine, Indianapolis, Indiana, United States of America; 5 Clem McDonald Center for Biomedical Informatics, Regenstreif Institute, Inc., Indianapolis, Indiana, United States of America; 6 Department of Clinical Pediatrics, Indiana University School of Medicine, Indianapolis, Indiana, United States of America; 7 Regenstrief Center for Health Equity Research, Eskenazi Health, Indianapolis, Indiana, United States of America; 8 Department of Biostatistics & Health Data Science, Indiana University School of Medicine, Indianapolis, Indiana, United States of America; 9 Center for Aging Research, Regenstreif Institute, Inc., Indianapolis, Indiana, United States of America; PLOS: Public Library of Science, UNITED KINGDOM OF GREAT BRITAIN AND NORTHERN IRELAND

## Abstract

**Introduction:**

Individuals with food insecurity are disproportionately burdened by hypertension (HTN) and type 2 diabetes and face greater barriers to self-managing these conditions.

**Methods:**

Food Resources and Kitchen Skills (FoRKS) is an ongoing 2-arm parallel randomized controlled trial (RCT) that will enroll 200 adults (35–75 y) with food insecurity and elevated systolic blood pressure (≥120 mmHg) at a large federally qualified health center (FQHC) network in Central Indiana. Blood pressure is measured using an ambulatory blood pressure monitoring (ABPM) device. The (FoRKS, N = 100) intervention integrates hypertension self-management education and support (SMES) with a home-delivered ingredient kit and cooking skills program (16 weeks). Enhanced Usual Care (EUC, N = 100) includes usual care services by the FQHC network, SMES classes (separate from FoRKS), and grocery assistance. This paper describes the protocol for this RCT that will: 1) test the efficacy of FoRKS compared to EUC for reducing systolic blood pressure using an intention to treat protocol, 2) identify behavior change levers (e.g., engagement, social support) and their associations with change in food insecurity, diet quality, and systolic blood pressure, 3) examine the maintenance of outcomes, and 4) assess cost-effectiveness.

**Conclusions:**

Establishing that a food insecurity and SMES intervention, compared to usual care services, is feasible in FQHCs and efficacious for improving blood pressure and related outcomes would have important public health implications. Understanding the behavior change levers of FoRKS that are associated with changes in health outcomes, whether these outcomes are maintained, and its cost-effectiveness will inform future efforts to address health disparities.

## Introduction

The World Health Organization defines social determinants of health (SDOH) as “conditions in the environment…that affect a wide range of health, functioning, and quality-of-life outcomes and risks [1]” Food insecurity—inconsistent access to a sufficient quantity of affordable and nutritious food [2]—is an adverse SDOH that is associated with health disparities among vulnerable minority populations [3, 4]. Food insecurity is associated with poor nutrition, which, in turn, is associated with about one-half of all cardiometabolic-related deaths among minority and less educated White adults [4]. Indeed, hypertension (HTN) and type 2 diabetes mellitus are as much as two times more prevalent among adults with food insecurity [4–6].

Self-management education and support (SMES) programs are used as a first-line, evidence-based approach for treating HTN and type 2 diabetes [7–9]. A major limitation of SMES programs is the lack of integration with social care. Integration of medical care and social care may be critical to address health disparities [10] Food-insecure conditions are associated with barriers to self-management of diet-sensitive chronic conditions [11, 12], and adults with food insecurity have been found to be less responsive to SMES interventions [13, 14]. One study that sampled over 1,200 study participants from food pantries across the United States (US) demonstrated that individuals with food insecurity and cardiometabolic disease sometimes faced trade-offs between medicine and food [15]. Authors of that study concluded that the effectiveness of SMES programs could be enhanced by directly and simultaneously addressing food insecurity [15].

To address food insecurity, it is important to consider the environmental and behavioral levers that may increase one’s access to affordable and nutritious food as well as the skills to prepare such food. Persons with food insecurity are more likely to live in communities with reduced or inconsistent access to affordable and nutritious food [16, 17], and to have limited access to lower cost groceries and cooking tools due to budget and transportation limitations [18].

Persons with food insecurity have also been shown to have lower general self-efficacy (e.g., I can generally manage to solve problems) and food-related self-efficacy [11, 19, 20], and lower food preparation, management, and cooking skills [19, 20]. Impacts of food resource and skill limitations include less nutritious food in the home and lower quality diets [16, 19]. Although large, high quality multisite trials have established that dietary change can have significant positive effects on blood pressure and blood glucose [21, 22], studies have yet to integrate SMES programs with programs that simultaneously address food resources and skills and rigorously assess their impacts on diet quality and health outcomes.

To address these gaps in practice and research, our team developed the Food Resources & Kitchen Skills (FoRKS) intervention. The FoRKS intervention is integrated with SMES programs and was designed for individuals with food insecurity and HTN (some of whom also have type 2 diabetes). FoRKS includes weekly home-delivered ingredient kits and home-based, hands-on cooking and nutrition classes led by experienced registered dietitians (RDs) who instill culinary skills (e.g., planning, preparation, and cooking skills) and enhance nutrition knowledge and food-related self-efficacy. The nutrition component is guided by a Mediterranean influenced diet—foods, particularly those high in polyphenols (e.g., certain vegetables, legumes, berries, nuts, seeds), that have been shown to have broad health-enhancing potential [23]. Diets rich in these foods improve metabolic function [24, 25] and blood pressure, combat oxidative stress, and improve lipid profiles [21, 23, 26, 27].

Findings from our pilot study on FoRKS demonstrated 87% attendance rate and significant improvements in food security and food-related self-efficacy [28]. However, that study was a one-arm pre-post trial with only 13 participants [28]. As a next step in this line of work, we designed a FoRKS randomized controlled trial (RCT). With funding from the National Institute on Minority Health and Health Disparities (R01 MD017961), the FoRKS RCT includes 4 specific aims: 1) test the efficacy of FoRKS for reducing blood pressure (and hemoglobin A1c (HbA1c) among participants with T2D); 2) identify behavior change levers and their associations with changes in food security, diet quality, and systolic blood pressure; 3) examine the maintenance of outcomes; and 4) assess the FoRKS intervention cost-effectiveness—all compared to EUC. We hypothesized that, compared to EUC, FoRKS will demonstrate greater reductions in systolic blood pressure and improvements in food-related social support, food self-efficacy, food security, diet quality, and food management skills. We also hypothesized that higher engagement will be associated with greater social support, self-efficacy, and food skill gains. This paper describes the protocol for the FoRKS RCT.

## Methods

**Fig 1:**
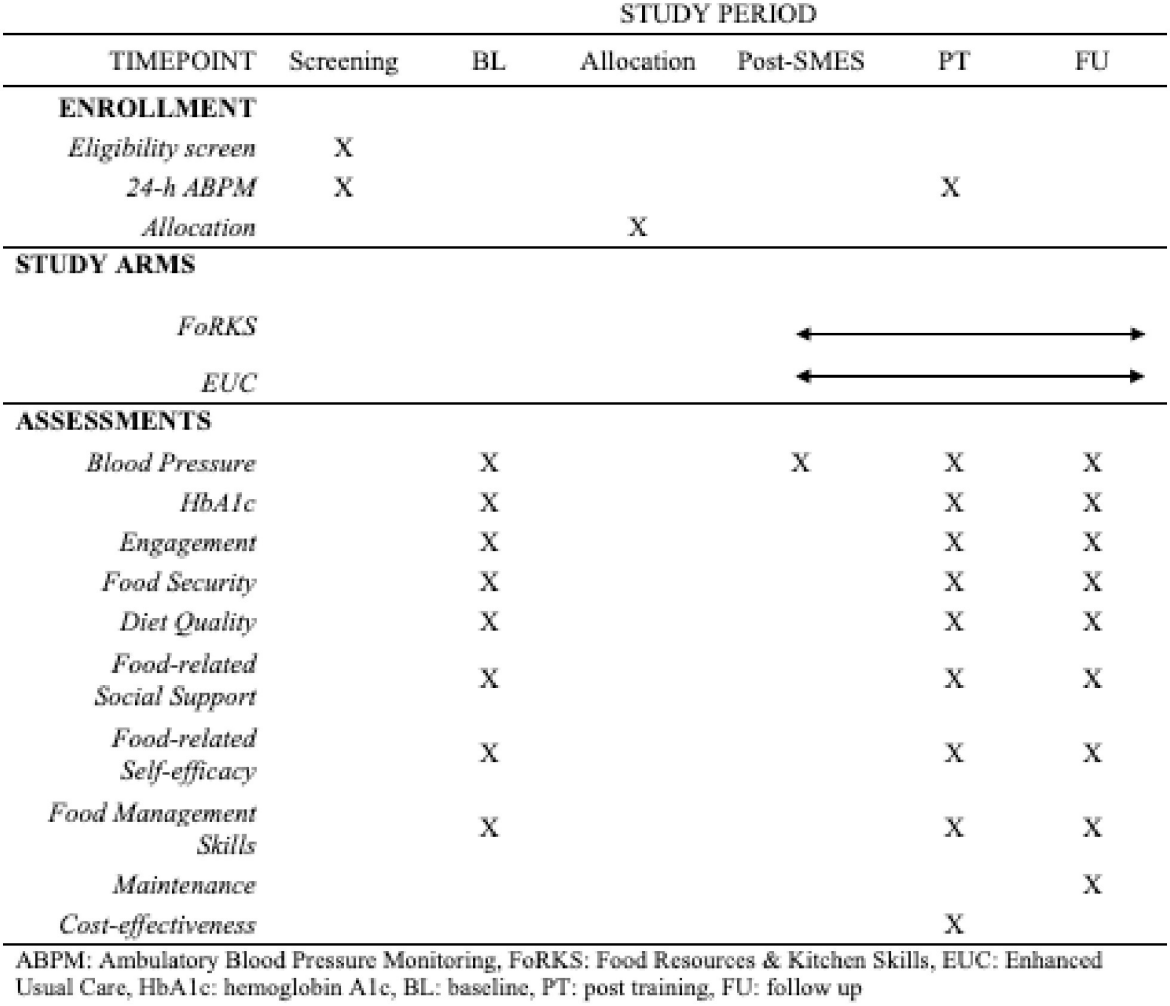
SPIRIT checklist.

This paper is guided by the SPIRIT framework (Standard Protocol Items: Recommendations for Interventional Trials) for developing study protocol papers [29, 30]. Per SPIRIT guidelines, the schedule of enrollment, interventions, and assessments are mapped in Fig 1.

### Study design and study setting

In this 2-arm parallel RCT, study participants are randomized to intervention or EUC in a 1:1 ratio. The study is conducted at Eskenazi Health’s Federally Qualified Health Centers (FQHCs). Eskenazi Health is one of the largest safety-net health systems in the US, providing primary care to over 100,000 unique adults each year through ten FQHCs located in medically underserved communities of Indianapolis, IN.

### Recruitment and eligibility

We were open to accrual on April 3, 2023 and initiated recruitment April 13, 2023. The anticipated recruitment end date is May 31, 2027. To recruit participants, we utilize the Eskenazi Health electronic medical record (EMR) system, provider referrals, and self-referrals. Potentially eligible adults are identified from the EMR via Regenstrief Data Services based on study-specific criteria. From this list of patients, initial study approach and eligibility screening are conducted by study personnel. Potentially eligible adults may also be identified by FQHC providers who conduct initial approach with basic study information. For interested patients, FQHC providers securely send contact information to study personnel to conduct eligibility screening. Eskenazi patients can also self-refer by completing a survey accessed with a Research Electronic Data Capture (REDCap)-generated quick response (QR) code or contacting the study team via phone or email.

Specific eligibility criteria are listed in Table 1. Briefly, we enroll midlife and older adults aged 35–75 years with normal cognition, food insecurity, and a mean systolic blood pressure ≥120 mmHg obtained from 24-hour ambulatory blood pressure monitoring (ABPM). Participants who do not meet eligibility criteria for the 24-hour ABPM are compensated $10 to conclude their participation.

**Table 1:** Study eligibility criteria.

*Inclusion Criteria*
1. Fluent in English
2. Marion County resident
3. Age 35–75 y
4. Ability to see and read street signs (self-report)
5. Stable housing with independent access to kitchen, including functional stove or hotplate, oven, refrigerator, and freezer (self-report)
6. Activity independence per Functional Activities Questionnaire (FAQ; <3 responses of "Require Assistance" and 0 responses of "Dependent")
7. Food insecurity per first two items of USDA 18-item survey with ≥ 1 response of “Often true” or “Sometimes true” [1) Within the past 12 months, you worried that your food would run out before you got the money to buy more; 2) Within the past 12 months, the food you bought just didn’t last and you didn’t have money to get more.] OR currently listed as food insecure in Eskenazi EMR; OR currently receiving SNAP benefits.
8. Normal cognition per Six-Item Screener (SIS; score ≥5)
9. Mean systolic blood pressure ≥120 mmHg from 24-h ABPM (from a minimum of 6 daytime readings and 2 nighttime readings)
*Exclusion Criteria*
1. Lives in nursing home
2. Diagnosis of dementia or Alzheimer disease or mild cognitive impairment; Parkinson disease; brain tumor/infection/surgery (within the last 10 y with residual symptoms and/or functional loss/deficit, such as impaired learning, memory, or communication); psychosis, schizophrenia, or bipolar disorder
3. ICD 10 code I11/hypertensive heart disease, ICD 10 code I12/hypertensive CKD, ICD 10 code I13/hypertensive heart disease and CKD, ICD 10 code I15, or ICD 10 code I16
4. Alcohol consumption ≥8 drinks per week for women, or ≥15 drinks per week for men
5. Drug use/abuse (excluding marijuana) per EMR
6. Moving out of area during study timeline
7. Scheduling conflicts with intervention schedule
8. Unwilling to use a touchscreen
9. Unwilling to be on video conferencing
10. Low communicative ability, functional status, or other disorders (examiner rated) that would interfere with interventions and assessments unable to provide informed consent
11. Unable to provide informed consent

FAQ: frequently asked questions, ABPM: ambulatory blood pressure monitoring; CKD: chronic kidney disease, ICD: international classification of diseases

### Ethics

All research personnel complete human participants training certification and annual HIPAA training. All participants provide written informed consent prior to participation in the study. This study has been approved by Indiana University’s Institutional Review Board and is registered at www.clinicaltrials.gov (NCT05856591).

We assign unique identifiers to track participants’ data and specimens to assure the privacy of participants and confidentiality of study data. To ensure privacy of enrolled participants, data collection occurs in the participant’s home, in a private exam room in the FQHC, or via telephone or video conference (S1 Appendix).

### Study procedures

**Fig 2:**
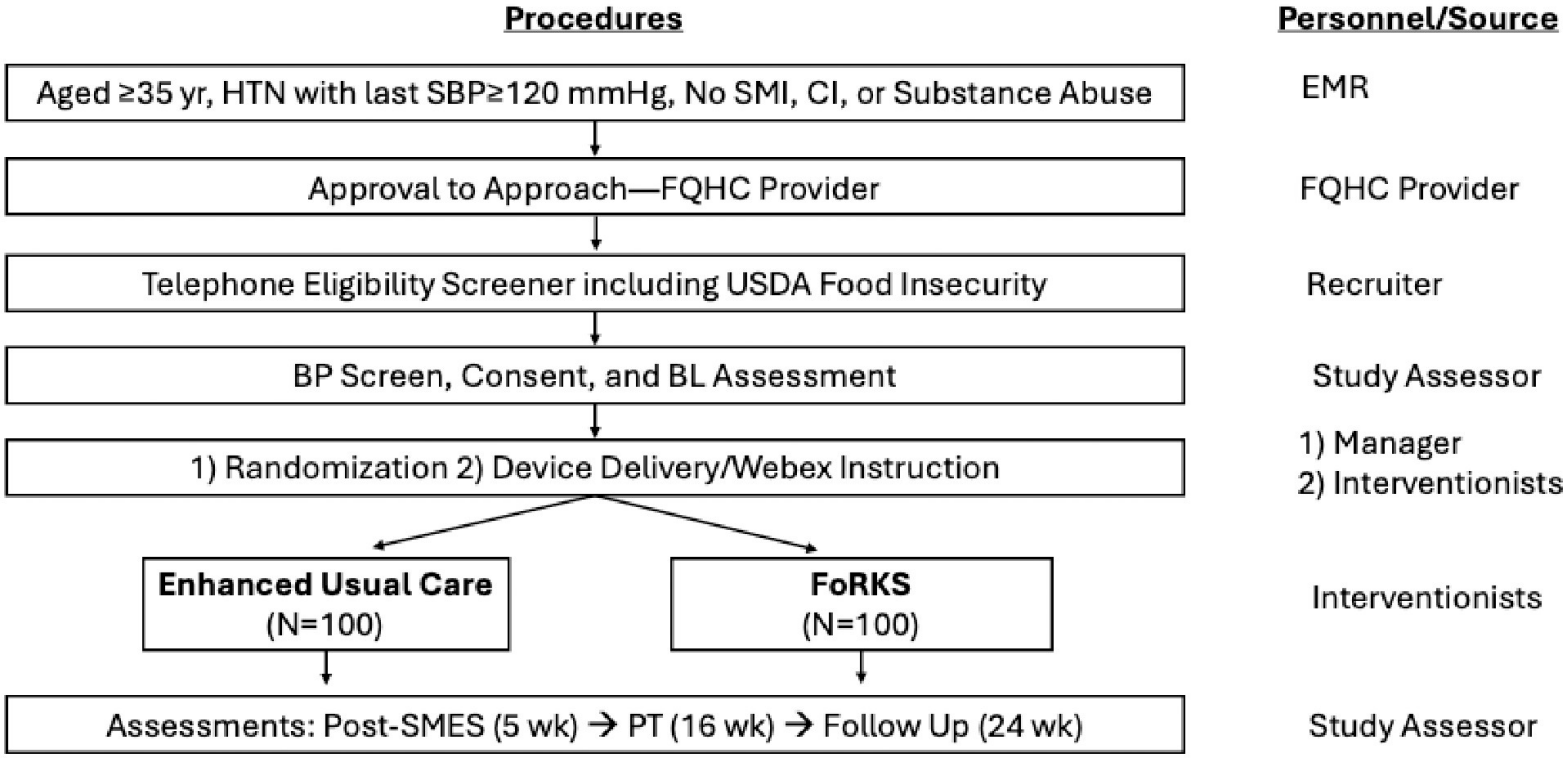
Flow chart of screening, assessment, randomization, and intervention. SBP: systolic blood pressure, SMI: severe mental illness, CI: cognitive impairment, FQHC: federally qualified health center, USDA: United States Department of Agriculture, BP: blood pressure, BL: baseline, SMES: self-management education and support, PT: post-training.

After the initial screening for eligibility, the study has four scheduled assessments: Baseline (BL; Week 0), post-SMES (Week 5), post-training (PT; Week 16), and/or follow-up (FU; Week 24), through a flow illustrated in Fig 2.

For screening, research personnel contact patients to communicate a scripted explanation of the project and confirm eligibility criteria. We enroll only one individual per household. Upon meeting the screening criteria, we proceed to the 24-hour ABPM for final eligibility determination. We use Spacelabs Healthcare (Snoqualmie, WA) monitors to measure ambulatory blood pressure three times per hour during potential participants’ normal daytime hours and two times per hour during their normal nighttime hours. Individuals meeting eligibility for average systolic blood pressure and successful number of ABPM readings during day and night are invited to participate in the project. Consented individuals are enrolled.

Treatment assignment occurs approximately two weeks before intervention for participants who have completed the BL assessment and confirmed availability for the next intervention start date. Participation occurs in replicates, or waves of cohorts, of 3–8 individuals in each study arm per replicate. Randomization is stratified by race (i.e., Black/White), sex, and type 2 diabetes status (based on EMR) to ensure balance in treatment assignments in key subgroups. Code for randomization is provided by the study statistician and implemented by data managers through the REDCap study database. When a participant is ready to be randomized, the study manager confirms the stratification factors for that participant and clicks the “Randomize” button to pull the next available assignment from the allocation sequence. Participants and providers are not blinded to the study due to the nature of the study, which assesses two behavioral interventions. Study personnel (i.e., Assessors) enroll the participants, and the study manager assigns the participants to interventions.

Outcome assessments are completed in the home, at the FQHCs, or by telephone, using REDCap direct data entry when appropriate. BL, post-SMES, PT, and/or FU assessments require about 90 minutes. The post-SMES visit differs from the others in that only blood pressure measurements are collected. Participants receive a $20 gift card for each assessment timepoint and a $5 gift card for each dietary recall questionnaire completed (up to 6 for a total of $30). Reloadable gift cards are distributed in person or mailed to the participant.

### Study arms

In both study arms, all participants have one-on-one meetings with a RD before group sessions to undergo individual assessments and personal behavior goal setting. Participants may meet with the RD either in person or via telephone to complete this assessment which gathers information on the participant’s usual diet and activity habits, barriers to health goals, and self-reported health confidence level. At the conclusion of each visit, the participant sets a personalized behavior goal. Discontinuing the interventions for a particular participant would occur in the event of unexpected participant risk or at the request of the participant. There are no plans to modify the intervention for an individual participant as the interventions are group-based.

#### Enhanced Usual Care

Participants randomized to EUC have access to existing usual primary care services. This usual care is enhanced by Eskenazi Health in the form of HTN SMES classes—an existing program endorsed by the Centers for Disease Control and Prevention and offered at Eskenazi Health to provide information and skills for managing HTN—and reloadable gift cards that can be used anywhere for grocery assistance. HTN SMES classes are led by Eskenazi Health RDs with assistance from primary care providers, pharmacists (PharmD), and health coaches. SMES classes are conducted 100% virtually via videoconference technologies (WebEx) once per week for about 60 minutes each for 5 weeks. Internet-enabled tablets are provided by Eskenazi Health to participants for accessing remote SMES classes, including WebEx training. HTN SMES content areas include the following:

- Introduction to HTN: Basic pathophysiology, risk factors
- Risk Reduction: Complication prevention, monitoring/measurement (group medical visit)
- Nutrition Part 1: Sodium reduction
- Nutrition Part 2: DASH diet
- Being Active: Physical activity recommendations and barriers discussion
- Taking Medications: Review of common blood pressure medications, barriers discussion
- Group Summary: Review via activity/game such as bingo.
- Recipes Demos: RD demonstrates a heart healthy recipe; participants are provided a ‘care package’ with food ingredients and teaching tools (e.g., measuring cups, a resistance band to reinforce and practice skills learned in class).

SMES classes also include patient participants from Eskenazi Health not enrolled in the study. Starting in Week 6, EUC participants receive weekly $10 gift cards for grocery assistance through Week 24 (total of $190); when possible, reloadable cards are used.

#### FoRKS intervention

**Fig 3:**
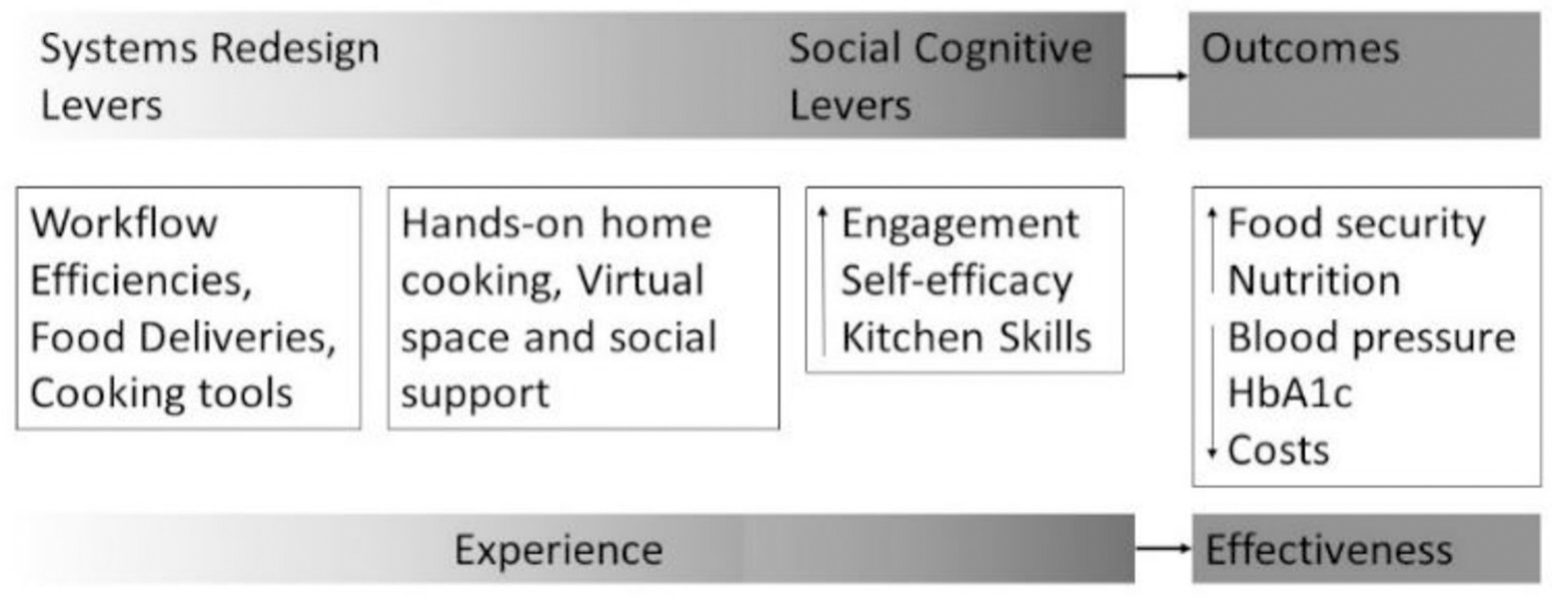
Theoretical framework. Figure previously published in Rivera et al. (2023) [28].

The FoRKS curriculum has been piloted and refined over the last 3 years by a transdisciplinary team including nutrition scientists, RDs, community members, human factors engineers, and behavioral scientists [28]. Social cognitive theory—a specific social learning theory—posits that environments designed to facilitate behavioral modeling and positive feedback from others will lead to self-efficacy gains [31]. Therefore, we conceptualized the intervention as environmental levers through which to facilitate behavioral engagement in social and experiential learning that over time improve food-related self-efficacy and budget-conscious food management skills (i.e., recipe selection, thrifty shopping, food planning and preparation, and cooking). These in turn we conceptualized as pathways to improve food security, diet quality, and health outcomes (Fig 3). Additionally, FoRKS is guided by Mediterranean diet principles. To reflect real world settings, we choose ingredients that are available at local grocery stores accessed by participants and consider participant preferences and food budget limitations. Participants randomized to FoRKS receive internet-enabled (via cellular data) tablet devices and tablet stands for video conferencing and remote participation in FoRKS classes. Training on the device is completed either in person or via phone.

In Weeks 1–5, participants randomized to FoRKS attend weekly HTN SMES classes separately from EUC participants. SMES classes include the EUC curriculum stated above and an introduction to the upcoming FoRKS intervention. In Weeks 6–16, FoRKS continues with home-delivered Mediterranean influenced ingredient kits (all meals ≤500 mg sodium), food management lessons, and hands-on remote cooking classes in participants’ own kitchen. Classes are led by RDs via Webex from physical space within an FQHC facility and, at times, from the RDs’ homes.

Class content may include a discussion of the day’s recipe (nutrition, ingredients) and needs for preparation (kitchen equipment, safe/sanitary space), hands-on cooking (instruction and demonstration of tools and various cooking techniques), lessons on budgeting, meal planning, and shopping, and a virtual group meal (sample the meal together and share thoughts about the food and lesson). These classes are held twice per week through Week 12, then only once per week during Weeks 13–16; weeks with holidays may have classes canceled in which case are rescheduled to be incorporated in future classes at the discretion of the RD. Classes are supported by study-provided cooking resources. Specifically, participants receive fresh, home-delivered ingredient kits weekly through Week 13. Additionally, each participant receives a set of kitchen tools and utensils to participate in cooking classes (e.g., cutting board, chef’s knife, paring knife, spatula, mixing spoon, measuring spoons, liquid and dry measuring cup, can opener, strainer, saucepan, skillet, mixing bowls, zester, vegetable peeler, and meat thermometer). During Week 14, in lieu of cooking class, RDs lead participants through a live-streamed local grocery store tour to incorporate lessons learned from previous weeks. During Weeks 15–16, participants are responsible for securing their own ingredients.

Engagement and satisfaction in classes are enhanced through intervention related incentives (e.g., apron and reusable grocery tote with study logo) and communication games interspersed with milestone activities (e.g., party hats and necklaces worn in session and graduation certificates, etc.). Every 2–3 weeks, participants connect with study personnel (via home visits, video conference, or phone calls) throughout the intervention setup and trial to provide feedback on their experience and receive guidance (if requested) on kitchen workspace preparation and organization. These key study personnel are familiar to study participants throughout the study and is an important aspect of engagement as they deliver foods, make pre-group reminder calls, and assist with login at the time of group classes.

In the maintenance period (Weeks 17 through 24), classes and ingredient kit deliveries end, but participants have continued access to recipes, including ingredient lists and shopping tips. FoRKS participants also receive $10 each week onto a reloadable gift card (like the EUC arm) for grocery assistance.

### Outcomes (Table 2)

**Table 2:** Study outcomes.

Specific Aim	Outcome	Instrument/Measurement
1	Systolic blood pressure	Omron series 10 device
	HbA1c	Seimens DCA Vantage Analyzer
2	Engagement	Participation (sessions completed)
		Observational video coding
		Classroom Engagement Inventory
	Social support	Social Support and Eating Habits Survey
	Food-related self-efficacy	Food-related self-efficacy questionnaire
	Food management skills	Plan, Shop, Save, & Cook Checklist
	Participant Experience	Qualitative 1-on-1 interviews
3	Maintenance (food security, nutrition, systolic blood pressure, HbA1c)	Dichotomized by percent change (Maintained/Improved vs. Worsened)
4	Cost-effectiveness	Cost Effective Analysis

All outcome measures are collected at BL, PT, and FU, except for blood pressure which was also collected post-SMES. All surveys are administered by research staff and are conducted in participant homes, or over the phone if needed.

#### Blood pressure and HbA1c (specific aim 1)

Systolic blood pressure is measured three times at each assessment point using standard procedures with an Omron series 10 device (OMRON Healthcare, Inc.; Hoffman Estates, IL); pulse pressure is calculated as the difference between systolic and diastolic blood pressure. We also measure 24-hour blood pressure at PT using the Spacelabs Healthcare ABPM device (Snoqualmie, WA). Appropriately sized cuffs are used with bladder sizes that encircle 80–100% of arm circumference and widths that are at least 40% of arm circumference. Ambulatory blood pressure is measured three times per hour during the participant’s normal daytime hours and two times per hour during the participant’s normal nighttime (i.e., sleep) hours. Resting heart rate is recorded by the Spacelabs device. HbA1c is assessed with 1 μL of whole blood from a fingerstick using a point-of-care analyzer (Seimens DCA Vantage Analyzer) at each assessment and is the only biospecimen collected. Manufacturer operation guidelines are followed for obtaining and analyzing samples.

#### Food security, diet quality, and behavior levers (specific aim 2)

Food security status is assessed using the 16-item Four Domain Food Insecurity Scale (4D-FIS) [32]. The 4D-FIS aligns conceptually with the USDA eligibility tool but has more items to cover four experiential domains (shortage of food, unsuitability of food and diet, uncertainty in access to food, and lack of control over food situation). Internal consistency of the 4D-FIS has been found to range from 0.69 to 0.90. Diet quality is assessed using the National Cancer Institute’s Automated Self-Administered 24-Hour Dietary Assessment Tool (ASA24; 2022 version) [33]. The ASA24 is an online 24-hour dietary recall tool that uses a multi-pass method and visual aids for foods, beverages, and portion sizes. The ASA24 is administered with assessor assistance when required and covers one day from midnight to midnight. For this study, we complete the recall for two nonconsecutive days at each assessment. Dietary intake data collected using the ASA24 is used to compute USDA Healthy Eating Index-2020 (HEI-2020) scores to quantify diet quality.

Regarding behavior levers, five intervention processes are measured: engagement, social support, food related self-efficacy, food management skills, and participant experience. Engagement is “the act of being occupied or involved with an external stimulus” [34] and is assessed in two ways: observed participation (total number of sessions attended and longest streak of consecutive classes attended, per person) and by a modified Classroom Engagement Inventory survey [35] administered about every 3 weeks. This structured questionnaire has 16 items that assess, over the prior 3 weeks, the participant’s affective engagement (e.g., “I feel interested”), behavioral engagement (e.g., “I actively participate in class discussions”), cognitive engagement (e.g., “I go back over things that I don’t understand”), and disengagement (e.g., “I let my mind wander”).

Social support is a multi-dimensional construct that refers to social relationships that promote well-being through emotional, informational, or instrumental channels [36], Social support is assessed by the Social Support and Eating Habits Survey [36], which has been widely used in weight loss trials and has shown good test-retest reliability (range 0.57 to 0.86) and high internal consistency (Cronbach’s alpha 0.80 to 0.87) [37, 38]. This 10-item scale captures friend and family support or sabotage of healthy food habits.

Food-related self-efficacy is assessed by a 9-item questionnaire of basic cooking self-efficacy, meal preparation, and meal planning. This scale has good test-retest reliability (range 0.46 to 0.91) and high internal consistency (Cronbach’s alpha 0.84 to 0.86) [39] and captures change over time in response to cooking lessons [40].

Food management skills are assessed using the approach taken in evaluating the SNAP-Education programs at the University of California Cooperative Extension [41] and the University of Kentucky [42] We administer the Plan, Shop, Save & Cook Checklist, a 7-item scale that includes five response options from Never to Always and contains items such as “how often do you plan meals ahead of time?”, “how often do you compare unit prices before buying food?”, and “how often do you shop with a grocery list?” (Cronbach’s alpha of 0.77) [41].

Participant experience is assessed through individual surveys covering satisfaction, usability, and implementation of the intervention. These occur approximately every three weeks during the intervention period (post-SMES through Week 16) and include Likert scale (1–5) questions regarding cooking class, delivered foods, and tablet experience.

#### Maintenance (specific aim 3)

Maintenance is assessed from PT to FU by the percent of FoRKS versus EUC participants who maintain improved food security, diet quality, and systolic blood pressure (and HbA1c among those with T2D at BL).

#### Cost-effectiveness (specific aim 4)

To estimate costs, participants complete a resource use questionnaire at PT and FU to assess their costs to participate in the interventions. These costs may include time off from work or dependent care associated with class participation. Cost of participation will be estimated by multiplying participants’ reported time by age-appropriate national wages from the Bureau of Labor Statistics (BLS). Second, we will compute intervention delivery costs, consisting of non-research fixed and variable costs of administering the intervention. Third, we will compute personnel costs, calculated from interventionists’ time logs and national median wages for RDs available online from the BLS. Fourth, space cost charges will be prorated for size of space and time in use from the clinic cost accounting system. Fifth, actual food, delivery, equipment, and tool costs will be computed. All costs will be adjusted for inflation using the Consumers’ Price Index from the BLS. The societal costs will be estimated by adding all the above listed costs to estimate the incremental costs from the societal perspective of the FoRKS intervention (excluding SMES costs).

### Sample size

The sample size was determined to ensure adequate power for testing the primary hypothesis regarding the efficacy of FoRKS on systolic blood pressure. Specifically, we hypothesized that in comparison to EUC, FoRKS participants will have lower systolic blood pressure (-6.0 mm Hg) at PT. The clinical difference of 6 mmHg is supported by a meta-analysis of 48 randomized trials and 344,716 participants that showed a reduction of greater than 5 mmHg in systolic blood pressure was associated with a 10% reduction in major CVD events regardless of baseline systolic blood pressure [43]. Thus, in this study, we use 6 mmHg as the size of detectable difference because of its accepted clinical significance as indicated in recent work [44–46].

### Data management

When possible, assessment data are directly entered into REDCap, a web-based secure relational data management system for research data. A data dictionary including each measurement item and definition for each item response are generated and used to build the REDCap database. The database includes internal error checking algorithms to prevent erroneous data entry or missing data. EMR and biomarker data are merged with other study data and stored on secure servers with access limited to data analysts working on this study and those meeting requirements for data sharing.

### Data analysis

The primary analyses will be carried out following an intention-to-treat (ITT) principle [47]. Per-protocol analyses will be performed secondarily as supplements to the ITT analyses [48]. We will use the trial data to determine the effects of FoRKS intervention on systolic blood pressure and, secondarily, glucose management. Mean systolic blood pressure at PT will be compared between the two treatment groups using a t-test. Mean HbA1c will be compared using the same test among individuals with type 2 diabetes at baseline. A similar analysis will be performed at FU to assess maintenance in outcomes. In addition to the main analysis described above, we will use mixed-effect models to assess the time course of the intervention effects by modeling repeatedly measured BP (BL, Post-SMES, PT, and FU) and HbA1c (BL, PT, FU); random subject effects will be included to account for the potential correlation among repeated measures contributed by the same subjects. These secondary analyses will allow us to control the effects of any participant characteristics that are not balanced.

We will examine behavior change levers that are associated with food security, diet quality, and systolic blood pressure. While parts of the analyses are exploratory, we are interested in testing hypotheses that FoRKS participants will report greater food-related social support and self-efficacy and food management skills at PT, and these gains will partially account for improved food security, diet quality, and systolic blood pressure in FoRKS participants relative to EUC. To analyze, we will first perform direct comparisons by using t-tests, but also regression analysis using mixed effect models. Specifically, we will enter repeated measures of food security, diet quality, and systolic blood pressure as dependent variables in the mixed effect models and test the effects of food-related social support and self-efficacy, and food management skills. Similarly, to evaluate the hypothesis that FoRKS participants showing greater learning engagement also experience greater social support, self-efficacy, and food management skill gains, we will use regression models to examine the association between learning engagement and social support, self-efficacy, and skill gains in FoRKS participants. We will explore whether SNAP participation modified intervention effects on food security, diet quality, or systolic blood pressure. We will also content-analyze participant experiences with the interventions to better understand themes such as barriers, facilitators, implementation issues, and cultural fit.

From PT to FU, we will determine and report the percent of FoRKS vs EUC participants who maintain improved food security, diet quality, systolic blood pressure, and HbA1c. We will dichotomize changes in these outcomes (maintained/improved outcome vs worsened outcome) between PT and FU. We will report the percentages of maintenance and improvement in each of the outcomes. We will also conduct logistic regression analyses to model the dichotomized outcomes and identify factors associated with improvement in each of the outcomes.

Finally, we will provide an initial indication of the cost effectiveness of FoRKS intervention. We will use the best practices of the Consolidated Health Economic Evaluation Reporting Standards [49, 50]. Specifically, we will estimate the cost and cost effectiveness of FoRKS relative to EUC. Using cost calculations for personnel, tools, equipment, delivery, and food, as well as participant costs, of the intervention, we will calculate average intervention costs as the sum of intervention costs divided by the number of subjects in the study group. We will then estimate incremental cost effectiveness ratios for blood pressure and HbA1c between study groups. In addition to the group differences, we will perform regression analyses on the cost effectiveness ratios to control for non-intervention factors known to influence systolic blood pressure and HbA1c (e.g., age).

### Data and safety monitoring

A data safety monitoring plan is executed by research personnel, including the project manager, investigators (including an internal safety officer), and a three-member Data Safety Monitoring Board (DSMB) that includes an external safety officer. The external safety officer for this trial is a physician researcher experienced in running intervention studies and understands the types and severity of risks associated with the FoRKS intervention. The DSMB also includes a nutrition scientist with trial experience and a biostatistician who is not to be otherwise affiliated with the study. The DSMB meets every 12 months. The project manager generates annual reports for study investigators, the external safety officer, and the DSMB. Review summary and recommendations from the DSMB are submitted to the IU IRB at the time of annual study renewal.

An internal (DR) safety officer reviews the reports sent by the study manager to determine whether there is any corrective action, trigger of an ad hoc review, or stopping rule violation that should be communicated to the principal investigator, the DSMB, the IU IRB, and the National Institute of Minority and Health Disparities. In addition, the safety officer may comment on whether the principal investigator needs to report any specific out of range data to the participant and/or her physician.

### Potential risks & harms

The primary risks associated with the study are from: (1) pain, soreness, or bruising from fingerstick, (2) fatigue, anxiety, stress, or embarrassment from self-report questionnaires, (3) pressure, itching, tightness, tingling, blistering/bumps/skin irritation or pain from the 24-hour ABPM, and (4) the loss of confidentiality or privacy. Foods in this trial are available in most conventional grocery stores and carry only ordinary everyday risk.

Assessed values that indicate the participant may be at very high risk are addressed immediately. For blood pressure, when a participant is found to have a systolic blood pressure of ≤90 mm Hg or ≥180 mm Hg or a diastolic blood pressure of ≥120 mm Hg, the assessor is to ask about shortness of breath, dizziness, lethargy, confusion, and chest pain symptoms. If any are present, with participant’s permission, the assessor calls for an ambulance. If not present, the blood pressure is rechecked. If still no symptoms, the participant is urged to call their primary care provider that day. In any case, the blood pressure value is shared with the primary care provider via the study’s internal safety officer. Similarly, any participant with an HbA1c value above 6.5% is given a recommendation to communicate with their primary care provider. For any participant with a value above 9%, our internal safety officer also shares the value and collection method directly with the primary care provider.

### Auditing

Interventionists are FQHC RDs who are trained in and observed facilitating the intervention classes by the lead RD designers (ED and MA) and according to the FoRKS protocol. No interventionist will act as study assessor and no assessor will act as an interventionist. Assessors complete a certification process prior to assessing enrolled participants. After certification, ongoing quality assurance checks continue throughout the study. Participants are asked not to share their study assignment with the assessors.

### Dissemination

We have a 4-part dissemination plan. First, we participate fully in scientific forums that allow us to deliver and receive information about this study and its potential contributions. This will be matched by publishing in scientific journals. Second, our media and communications staff within Regenstrief Institute, Inc., Indiana University School of Medicine, and Indiana University School of Public Health work to distribute news of study startup, enrollment opportunities, and study findings through social media and traditional print media. Third, the FoRKS intervention is potentially scalable through Internet-enabled devices and the nation’s growing meal delivery systems. This makes for efficient and potentially rapid dissemination. Fourth, we will present the study’s findings at America’s Essential Hospitals conference with the CEO of Eskenazi Health. America’s Essential Hospitals represent the nation’s safety-net health and primary care systems. This network reaches all corners of the nation and has multiple joint initiatives and is a very effective dissemination network and complementary to the usual academic and media channels with its access to safety-net systems and communities. Finally, our team will develop system design and workflow requirements that can be included in dissemination materials to safety-net and other health systems.

We anticipate that we would apply for and receive local IRB approval to construct a de-identified dataset that could be made available to other researchers. For some data, this will also require approval of participating health care systems that contribute data to the regional health information exchange.

### Protocol amendments

Any changes to the protocol are submitted for approval through the IRB after communication with the DSMB.

## Discussion

The FoRKS intervention was designed with and for a large safety-net health system whose leaders have enthusiasm for the potential of FoRKS to address an important social need and related health disparities. We recognize, however, that SDOH root causes (e.g., generational poverty and historical racism) lie behind some of the excesses in blood pressure and glucose levels among persons with food insecurity [49]. We also recognize that individual-level interventions, even when, like FoRKS, target the social environment, are not in themselves sufficient to address all unmet health related social needs or health disparities.

COVID-19 has accelerated telehealth and Eskenazi Health now delivers SMES over Webex videoconference. A recent report showed primary care visits across 41 FQHCs declined just 6.5% from prior to during the pandemic due to telehealth adoption, which was nearly one-half of visits [51]. On observation, we have seen that many safety-net patients have a phone with a data plan. However, as we do not want device or Internet access disparities to be part of the intervention evaluated, we provide a tablet and cellular data plan for all study participants. Lack of Internet access is an adverse SDOH and a source of disparities. A positive trial here could, to a small degree, support the case of broadband access as a public good vital to all.

Given pervasive challenges among underrepresented minority populations in biomedical research, several strategies are used to ensure adequate and diverse recruitment. Among those enrolled we monitor engagement in detail as described. For both recruitment and retention, we monitor issues and work to improve quality where needed, e.g., by increasing participation incentives or simplifying recruitment workload on research staff and patients.

## Conclusion

This study, to our knowledge, will be the first to rigorously test the efficacy of an intervention that integrates SMES programs for HTN (and type 2 diabetes) and a food insecurity and food management skills program among a population with high risk of developing cardiovascular diseases. Given the real world nature of the study, there is strong potential for implementation locally and at scale to reduce health disparities among populations with food insecurity and HTN.

## Supporting information

S1 Appendix Informed consent materials. (DOCX)

S1 File (DOCX)

S1 Checklist SPIRIT 2013 checklist: Recommended items to address in a clinical trial protocol and related documents*. (DOC)
