# Supplementary material for: The Food Resources and Kitchen Skills intervention: Protocol of a randomized controlled trial
Source: PLoS One. 2025 Feb 6;20(2):e0314275. doi: 10.1371/journal.pone.0314275 (PMC11801624; doi:10.1371/journal.pone.0314275)
Supplement: S1 Appendix — (DOCX) [file pone.0314275.s001.docx]

# INDIANA UNIVERSITY INFORMED CONSENT STATEMENT AND AUTHORIZATION FOR RESEARCH

**Delivering Food Resources & Kitchen Skills (FoRKS) to Adults with**

**Food Insecurity and Hypertension: An RCT**

**IRB Protocol #16773**

## ABOUT THIS RESEARCH

You are being asked to participate in a research study. Research is done to answer important questions which might help change or improve the way we do things in the future.

This consent and authorization form will give you information about this study. It is your choice whether or not you want to be in this research study. Please read this form, and ask any questions you have, before agreeing to be in this study.

## STUDY SUMMARY

Participation in this study will last approximately 6-7 months. Activities vary as listed in the Visit Timeline below. Through participation in this study, you will learn ways to manage high blood pressure, gain social contact through group sessions, and may gain greater access to food. Risks of participation are minimal, ranging from feeling tired or stressed during the group sessions and study visits, pain or bruising from the fingerstick or blood pressure cuff, and loss of confidentiality as other group participants will know who you are.


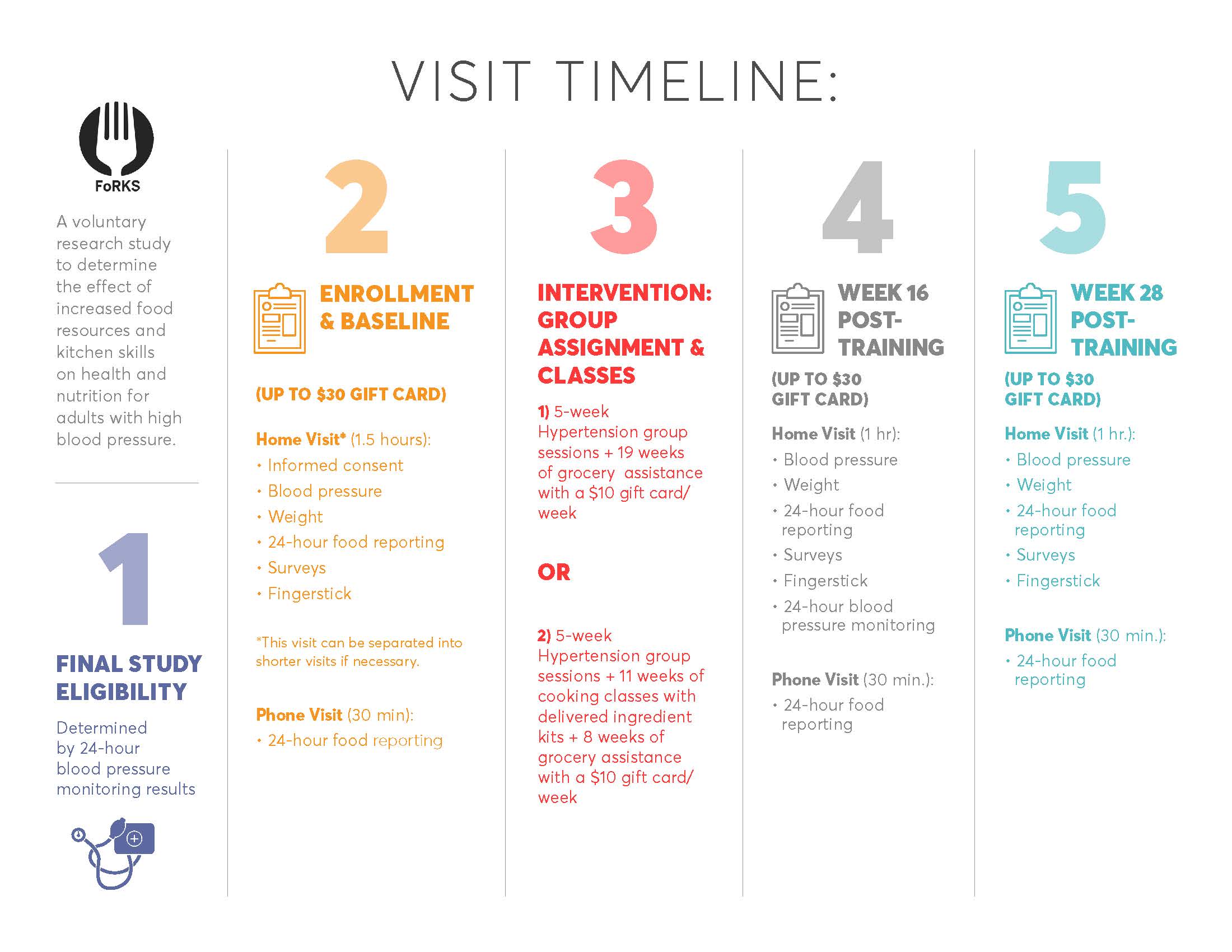


## WHY IS THIS STUDY BEING DONE?

The purpose of this study is to determine the impact of a home-delivered foods and kitchen skills program on health and nutrition.

We are asking you if you want to be in this study because you are an adult in the Eskenazi Health system who had an average systolic blood pressure of 120 mm Hg or greater during the 24-hour ambulatory blood pressure monitoring you completed at home. You may also have expressed concern about your access to enough food at an Eskenazi visit.

The study is being conducted by Dr. Daniel Clark of Indiana University Center for Aging Research and Dr. Richard Holden of Health and Wellness Design at Indiana University. It is funded by National Institute on Minority Health and Health Disparities.

## WHAT WILL HAPPEN DURING THE STUDY?

If you agree to participate, you will be in this study for approximately 6-7 months.

After confirming you meet all study criteria, your Baseline visit will continue with the following:

- **In your home**, review and sign informed consent if you want to participate. After consent, we will measure your weight and blood pressure, ask you to complete several questionnaires about your health, mood, diet, food shopping, cooking habits, and demographics, complete an activity using a nutrition label, and report the foods you ate over the last 24-hour period. An experienced technician will perform a fingerstick with a sterile lancing device to obtain 1 droplet of blood. This sample will be used to measure sugar levels in the blood. This home visit will take about 1 ½ hours to complete.
- **At least one day later, over the phone**, you will be asked to report the foods you ate over the last 24-hour period. This telephone visit will take about 30 minutes to complete.

After Baseline is complete, you will be randomly assigned to participate in 1) Hypertension group **OR** 2) Hypertension group *plus* Extended Training. You will be randomly assigned (like flipping a coin) to one of these two groups; neither you nor the researcher can choose whether you will be placed in Extended Group Training.

Before starting group, you will be contacted by an Eskenazi dietitian to complete an enrollment appointment. This can be completed in person at an Eskenazi Health Center or by phone and may take 30-60 minutes.

If you are unable to start group due to changes in your schedule or health after being randomly assigned, you will be offered the chance to participate in a future group. You may be asked to repeat the 24-hour blood pressure monitoring, Baseline Visit, and dietitian enrollment appointment prior to group participation.

**Hypertension Group.** You will be scheduled for group sessions once a week for 5 weeks. These sessions will include education about high blood pressure and how it can be managed through medications, physical activity and nutrition.

Sessions are about one-hour each and led by an Eskenazi dietician either in person at an Eskenazi clinic, or via video conference with up to 20 other participants. Some participants may not be part of the FoRKS study; they may be participants of a similar research study or Eskenazi patients with a doctor referral for this group.

Communication via video conference is like live, interactive television over the internet. The instructor and all group participants will be able to hear and see one another. The dietician in charge of your group will give you instructions on where and how to join these sessions. Eskenazi will loan you a device (smart phone or tablet) to join these virtual sessions if you need one; you will be asked to sign an Equipment Use Agreement.

Near the end of these 5 weeks, we will measure your blood pressure during a quick visit to your home. If you were loaned a device from Eskenazi, we will collect it from you at this visit. If the tablet is not returned or lost, service to the tablet will be deactivated.

**Extended Group Training.** After Hypertension group sessions are complete, participants assigned to Extended Training will continue study participation with 11 weeks of live cooking/instructional classes. Again, some participants in this group may be participants of a similar research study instead of FoRKS.

If you are assigned to this group, you will receive a tablet device with a stand and internet access, and training on how to use it. You will be asked to sign an Equipment Use Agreement. If you have problems with the equipment, our study team is available to assist you over the phone or at your home if necessary. This equipment will be collected from you after training is complete. If the tablet is not returned or lost, service to the tablet will be deactivated.

Weeks 6 through 12 include:

- 2 cooking and/or instructional classes per week. These classes are approximately 1.5 hours each and led by an Eskenazi dietician via video conference with up to 14 other participants. There may be additional project personnel monitoring these video conferences and sessions may be recorded.
  - You will use ingredient kits delivered to your home to participate in these cooking classes. Following a recipe created for that class, the dietician will guide you in preparing the meal in your own kitchen. Classes will also include food management, nutritional lessons, and grocery budgeting tips.
  - During weeks with holidays, classes may be cancelled or rescheduled. These changes will be communicated to you by the dietician.

Weeks 13 through 16 include:

- 1 cooking and/or instructional class per week via video conference (as described above).
- You will receive an ingredient kit for Week 13, and be responsible for securing your own ingredients for the last 2 weeks.

The study team may connect with you periodically (via home visits, video conference, or phone calls) throughout the training to provide guidance on kitchen workspace prep and organization.

Approximately every 3 weeks you will be asked to provide feedback about your experience with the training.

**Post-training and Follow-Up Visits for all participants:** Around Week 16, and again around Week 24, you will be asked to complete study visits identical to the Baseline visit. Again, activities will be completed in your home and by phone, and on two different days you will be asked to report the foods you ate over the last 24-hour period.

For the Week 16 visit only, you will be asked to wear an automatic blood pressure monitor for 24 hours (one full day and night). This is the same small, portable device that automatically checks your blood pressure that you wore on your arm to determine eligibility for this project. The device will automatically measure your blood pressure every 20 minutes during day-time hours, and every 30 minutes during night-time hours. The technician will assist you with placement of this device and instruct you on how to use it, and will return to pick it up after 24 hrs.

If you participate in this study, we may learn things about you from the study procedures that could be important or interesting to you. Depending on the information, you might need to meet with professionals with expertise to help you decide what to do with the information. We do not have money or funds available to cover the costs of any follow-up consultations or actions. Any information that might be immediately critical to your health (e.g., high blood pressure, high blood sugar, and related symptoms) will be shared with you or your health care provider.

## WHAT ARE THE RISKS OF TAKING PART IN THE STUDY?

This study involves minimal risks. While participating in the study, the potential risks are:

1. You may feel tired or stressed during the group classes and study visits. You can tell the researcher that you do not care to answer a question or do a task or that you want to take a break.
2. You may have pain, soreness, and bruising from the fingerstick. To minimize these problems, blood will be obtained by trained staff using sterile procedures.
3. You may feel pressure, itching, tightness, tingling, blistering, bumps or pain from the device that automatically measures your blood pressure. You may experience skin irritation from the tubing or nylon strap that goes behind your neck. You may experience bruising or disrupted sleep. To minimize these symptoms, the automatic blood pressure monitor will be placed by trained staff who will select a cuff that is the proper size for your arm. Blood pressure cuffs and tubing will be cleaned after every use. It is recommended you wear clothing that can serve as a protective barrier between the skin, blood pressure cuff, tubing, and nylon strap to reduce the risk of some of these symptoms.  To minimize discomfort and increase the chance of a successful blood pressure reading, be as still as possible during each measurement. While sleeping, avoid laying on the arm that has the blood pressure cuff. If you remove the cuff from your arm and replace it on your own, you may experience other side effects we cannot predict.
4. The Hypertension Group sessions and Extended Group Training are conducted in small groups, so other people will see your face and know who you are in this project.
5. There is a potential risk of loss of confidentiality. We store background and health information about you on our computers and there is a small risk that that information could be exposed accidently to people outside the study. To guard against this, project documents are labeled with code numbers, not first or last names, and project documents are stored in secure locations accessible only to the study team.

## WHO WILL PAY FOR MY TREATMENT IF I AM INJURED?

If you are injured as a result of participating in this study, you will be responsible for seeking medical care and for the expenses associated with any care received. Costs not covered by your health care insurer will be your responsibility. Also, it is your responsibility to determine the extent of your health care coverage. No money or funds are set aside to pay for these types of injuries. However, you are not giving up any legal rights or benefits to which you are otherwise entitled by signing this Informed Consent form.

**WHAT ARE THE BENEFITS OF TAKING PART IN THE STUDY?**

You may gain an increased awareness of healthy and unhealthy activities through participation in this project. You will also gain social contact through class participation and feedback on your blood pressure and weight to aid in self-management of high blood pressure. You may also gain greater access to food and resources and improve your kitchen skills if you are assigned to the Extended Training Group.

## WILL I BE PAID FOR PARTICIPATION?

You will receive payment for taking part in this study in the form of gift cards which will be given to you in-person or mailed to you at the completion of the visit. When possible, reloadable gift cards will be used.

You will receive a $20 gift card when you complete your Baseline visit, a $20 gift card when you complete your Post-Training visit at 16 weeks, and a $20 gift card when you complete your Follow-Up visit at 24 weeks.

You will receive a $5 gift card for every 24-hour food report you complete, which is $30 total throughout the study. The earned value may be added to the respective study visit gift cards.

In total, you have the potential to earn up to $90 in gift cards for completing all study procedures.

If you are NOT randomized to Extended Group Training: You will receive a $10 gift card per week for 19 weeks after Hypertension Group sessions are complete to assist with food costs.

If you are randomized to Extended Group Training: You will receive a set of kitchen tools and utensils for participation in cooking classes that you can keep at the end of the study. You will also receive a $10 gift card per week for 8 weeks after the cooking classes end in Week 16 to assist with food costs.

## WILL IT COST ME ANYTHING TO PARTICIPATE?

There is no cost to you for taking part in this study. However, if you are randomized to Extended Group Training, you will be responsible for purchasing your own ingredients for participating in Weeks 15 and 16 cooking classes.

## HOW WILL MY INFORMATION BE USED?

The study team will collect information about you from your medical records. This information, some of which may identify you, may be used for research-related purposes. Your records may be accessed to ensure you meet the criteria to be in this study, to gather information about your medical history to include in the research data, to check on your health in the future to help answer our research questions, or to inspect and/or copy your research records for quality assurance and data analysis. If you experience an adverse event during your participation, such as a hospitalization or injury, your medical records may be accessed for reporting purposes and to determine your continuation in the study.

The information released and used for this research will include:

- Information provided by you
- Medical history / diagnoses / treatment
- Medications
- Consultations
- Laboratory / diagnostic tests

If you agree to participate, you authorize the following to disclose your medical record information:

- - Eskenazi Health
  - Eskenazi Health Physicians
  - Indiana University Health
  - Other, not listed above: ______________________________________________________________

The following individuals and organizations may receive or use your identifiable health information:

- The researchers and research staff conducting the study
- The Institutional Review Boards (IRB) or its designees that review this study
- Indiana University
- Regenstrief Institute
- US government or agencies as required by law
- Data safety monitoring boards and others authorized to monitor the conduct of the study
- State or Federal agencies with research oversight responsibilities, including but not limited to:
  - Office for Human Research Protections (OHRP)
  - National Institutes of Health (NIH)

Information collected for this study may be used for other research studies or shared with other researchers for future research. If this happens, information that could identify you, such as your name and other identifiers, will be removed before any information is shared. Since identifying information will be removed, we will not ask for your additional consent.

A description of this clinical trial will be available on [ClinicalTrials.gov](http://clinicaltrials.gov/), as required by U.S. law. This website will not include information that can identify you. At most, the website will include a summary of the results. You can search this website at any time.

## HOW WILL MY INFORMATION BE PROTECTED?

Every effort will be made to keep your personal information confidential, but we cannot guarantee absolute confidentiality. No information which could identify you will be shared in publications about this study. Video recordings of the cooking classes will be stored on a secure Indiana University Server accessible only to FoRKS researchers for analysis after the study. Your personal information may be shared outside the research study if required by law and/or to individuals or organizations that oversee the conduct of research studies and these individuals or organizations may not be held to the same legal privacy standards as are doctors and hospitals.

This research is covered by a Certificate of Confidentiality from the National Institutes of Health. This means that the researchers cannot release or use any information, documents, or specimens that could identify you in any legal action or lawsuit unless you say it is okay.

However, there are some types of sharing the Certificate does not apply to. The Certificate does not stop reporting required by federal, state, or local laws, such as reporting of child or elder abuse, some communicable diseases, and threats to harm yourself or others. The Certificate does not stop a government agency who is funding research from checking records or evaluating programs. The Certificate also does not prevent your information from being used for other research when allowed by federal regulations.

Researchers may release information about you when you say it is okay. For example, you may still give them permission to release information to insurers, medical providers, or others not connected with the research.

## WHO SHOULD I CALL WITH QUESTIONS OR PROBLEMS?

For questions about the study or a research-related injury, contact the researcher, Dr. Daniel Clark at 317-963-7301. After business hours, please call the Eskenazi Switchboard at 317-880-0000 and ask for the attending physician.

For questions about your rights as a research participant, to discuss problems, complaints, or concerns about a research study, or to obtain information or to offer input, please contact the IU Human Research Protection Program office at 800-696-2949 or at [irb@iu.edu](mailto:irb@iu.edu).

## WHAT IF I DO NOT PARTICIPATE OR CHANGE MY MIND?

After reviewing this form and having your questions answered, you may decide to sign this form and participate in the study. Or, you may choose not to participate in the study. This decision is up to you. If you choose not to participate in this study or change your mind after signing this document, it will not affect your usual medical care or treatment or relationship with Eskenazi Health or Indiana University.

If you change your mind and decide to leave the study in the future, you should contact the study team immediately. If you were randomized to Extended Group Training, food deliveries will end and study equipment will be collected from you.

If you choose to withdraw your authorization for use and disclosure of your protected health information, you must do so in writing by notifying Dr. Daniel Clark, C/O Lyndsi Moser, 355 W 16^th^ Street, Suite 2800, Indianapolis, IN 46202. If you withdraw your authorization, you will not be able to continue in this study. However, even if you cancel this authorization, the research team, research sponsor(s), and/or the research organizations may still use information about you that was collected as part of the research project between the date you signed this document and the date you cancelled this authorization. This is to protect the quality of the research results. Otherwise, this authorization remains valid until the research ends and required monitoring of the study has been completed.

The researchers may stop your participation in the study even if you do not want to stop if you decline to participate in the study activities, or if you fail to communicate with or mistreat study staff. The investigator may also stop your participation in the project if you become extremely anxious or disruptive to other participants; this will be done to protect your health as well as the training experience for the other participants.

## Agreement to be Contacted by Email

We would like to communicate with you about this study by email. We might use email to send you reminders about upcoming visits or appointments, check on how you are doing, or tell you about the progress of the research.

Email is not a secure method of communication. The information sent over email, which may include sensitive or personal information, such as protected health information, could be accessed or read by someone other than you. If you would like us to communicate with you via email, please initial the lines below and provide the email address(es) you would like us to use.

______ I authorize the researchers to send me emails related to this research study

Email address for this communication: __________________________________

You can still participate in this study even if you do not want us to contact you by email.

## PARTICIPANT’S CONSENT AND AUTHORIZATION

In consideration of all of the above, I agree to participate in this research study. I will be given a copy of this document to keep for my records.

**Participant’s Printed Name:**

**Participant’s Signature**: **Date**: ________________

(must be dated by the subject)

**Participant’s Address:** _______________________________________________

(Street)

_______________________________________________

(City, State, Zip)

**Printed Name of Person Obtaining Consent:**

**Signature of Person Obtaining Consent**: _______**Date**: __________
