# Supplementary material for: The Food Resources and Kitchen Skills intervention: Protocol of a randomized controlled trial
Source: PLoS One. 2025 Feb 6;20(2):e0314275. doi: 10.1371/journal.pone.0314275 (PMC11801624; doi:10.1371/journal.pone.0314275)
Supplement: S1 File — (DOCX) [file pone.0314275.s002.docx]

**Delivering Food Resources & Kitchen Skills (FoRKS) to Adults with Food Insecurity and Hypertension: An RCT**

## Dr. Daniel Clark, PhD

Indiana University Center for Aging Research

Regenstrief Institute, Inc.

Indianapolis, IN

daniclar@iu.edu

(317) 274-9292

## Dr. Richard Holden, PhD

## Indiana University

## Health and Wellness Design

## Bloomington, IN

## [rjholden@iu.edu](mailto:rjholden@iu.edu)

## 812-856-1965

**Support Provided by:**

National Institute of Health

National Institute of Minority Health and Health Disparities

R01 MD017961

**Protocol Version: 01/10/2024**

## Table of Contents:

1. **Rationale & Objectives**
2. **Eligibility Criteria**
3. **Study Design**
4. **Intervention**
5. **Measures/Outcomes**
6. **Statistical Considerations**

**Abbreviations:**

| 4D-FIS | Four Domain Food Insecurity Scale |
| --- | --- |
| ABPM | Ambulatory blood pressure monitor |
| ASA-24 | Automated Self-Administered 24-hour (ASA24®) Dietary Assessment Tool |
| BL | Baseline |
| BP | Blood pressure |
| CDC | Center for Disease Control |
| CEA | Cost Effectiveness Analysis |
| DASH | Dietary Approaches to Stop Hypertension |
| DM2 | Diabetes Mellitus Type 2 |
| EDTA | Ethylenediaminetetraacetic acid |
| EMR | Electronic Medical Record |
| EUC | Enhanced Usual Care (control arm) |
| EV | Eligibility Visit |
| FAQ | Functional Activities Questionnaire |
| FoRKS | Food Resources & Kitchen Skills (intervention arm) |
| FQHC | Federally-Qualified Health Center |
| FU | Follow-up |
| HTN | Hypertension |
| NHANES | National Health and Nutrition Examination Survey |
| NHB | Non-Hispanic Black |
| NHW | Non-Hispanic White |
| NIH | National Institutes of Health |
| NVS | Newest Vital Sign |
| PT | Post-training |
| RCT | Randomized Controlled Trial |
| RD | Registered Dietician |
| SDOH | Social Determinant of Health |
| SMES | Self-Management Education and Support |
| SNAP | Supplemental Nutrition Assistance Program |
| SPB | Systolic blood pressure |
| USDA | United States Department of Agriculture |

1. **Rationale & Objectives**

As an adverse social determinant of health (SDOH), food insecurity is inextricably linked to health disparities. Even before the pandemic, over one in five non-Hispanic Black (NHB) and one in twelve non-Hispanic White (NHW) households struggled to meet even basic food needs. Among adults with food insecurity, conditions such as hypertension (HTN) and type 2 Diabetes (DM2) are as much as two times more prevalent. This evidence has led health experts and professional societies to recommend the development and testing of interventions to directly address this SDOH.

This project is an NIH Stage III behavioral randomized controlled trial to evaluate nutritious food delivery and cooking classes (FoRKS) versus enhanced usual care (EUC). This project seeks to evaluate the potential for hands-on training in food management skills to create lasting improvements in food security, self-efficacy, nutrition, and risk factor reduction in persons with prevalent chronic disease.

EUC consists of SDOH screening, referrals to food pantries, assistance enrolling in food programs (e.g., SNAP), and access to CDC-approved HTN Self-Management Education Series (SMES) and, if applicable, DM2 SMES. *In addition to the EUC offerings*, participants randomized to FoRKS will receive home-delivered ingredient kits and Webex cooking classes with embedded lessons in kitchen organization, tool use, nutrition, budgeting, and shopping.

Specific Aims and Hypotheses:

1. **Primary Aim.** Determine the effects of FoRKS on disease management.

Effectiveness Hypothesis: Compared to EUC at PT, FoRKS participants will have lower mean systolic blood pressure (SPB) (-5.0 mm Hg). Among those with both DM2, we will secondarily examine FoRKS’ effect in lowering HbA_1c_.

1. **Intervention Processes**. Identify behavior change levers and their associations with change in food security, nutrition, and SBP.

Intervention Process Hypotheses: 2.1) FoRKS participants will report greater food-related social support and self-efficacy, and food management skills at PT and 2.2) these gains will partially account for better food security, nutrition, and SBP relative to EUC. 2.3) Higher learning engagement will be associated with greater social support, self-efficacy, and food skill gains among FoRKS participants.

1. **Maintenance.** From PT to FU, determine the percent of FoRKS versus EUC participants who maintain improved food security, nutrition, and SPB (and/or HbA_1c_ if applicable).
2. **Reach and Cost.** Assess reach and intervention cost-effectiveness in preliminary economic analyses.
3. **Eligibility Criteria**

The target population is midlife adults aged 35-75 years with normal cognition, food insecurity, and a recent systolic BP ≥120 mm Hg.

We will enroll only one individual per household.

**Inclusion Criteria:**

Fluent in English

Marion County resident

35-75 years

Systolic BP of ≥120 in prior 12 months from a primary care visit

Ability to see and read street signs (self report)

Stable housing with independent access to kitchen, including functional stove or hotplate, oven, refrigerator, and freezer (self report)

Activity independence per functional activities questionnaire (FAQ; <3 responses of "Require Assistance" and 0 responses of "Dependent")

Food insecurity per first two items of USDA 18-item survey with ≥ 1 response of “Often true” or “Sometimes true” [1) Within the past 12 months, you worried that your food would run out before you got the money to buy more; 2) Within the past 12 months, the food you bought just didn’t last and you didn’t have money to get more.] OR currently listed as food insecure in Eskenazi EMR; OR currently receiving SNAP benefits.

Normal cognition per six-item screener (SIS; score of ≥ 5)

1. At Eligibility Visit: Mean systolic BP of ≥120 from 24-hour ABPM (from a minimum of 6 daytime readings and 2 nighttime readings) [NOTE: ABPM from IRB#16547 can be used to satisfy this requirement. See details under “Study Design” section.]

**Exclusion Criteria:**

1. lives in nursing home
2. diagnosis of dementia or Alzheimer disease or mild cognitive impairment; Parkinson disease; brain tumor/infection/surgery (within the last 10 years with residual symptoms and/or functional loss/deficit, such as impaired learning, memory, or communication); psychosis, schizophrenia, or bipolar disorder
3. ICD 10 code I11/hypertensive heart disease, ICD 10 code I12/hypertensive CKD, ICD 10 code I13/hypertensive heart disease and CKD, ICD 10 code I15, or ICD 10 code I16
4. alcohol consumption ≥ 8 drinks per week for women, or ≥15 drinks per week for men
5. drug use/abuse (excluding marijuana) per EMR
6. moving out of area during study timeline
7. scheduling conflicts with intervention schedule
8. unwilling to use a touchscreen
9. unwilling to be on video conferencing
10. low communicative ability, functional status, or other disorders (examiner rated) that would interfere with interventions and assessments
11. unable to provide informed consent

1. **Study Design**

The study will operate out of Eskenazi Health’s FQHCs.

Figure 2 shows the two-arm, parallel RCT design of the study.


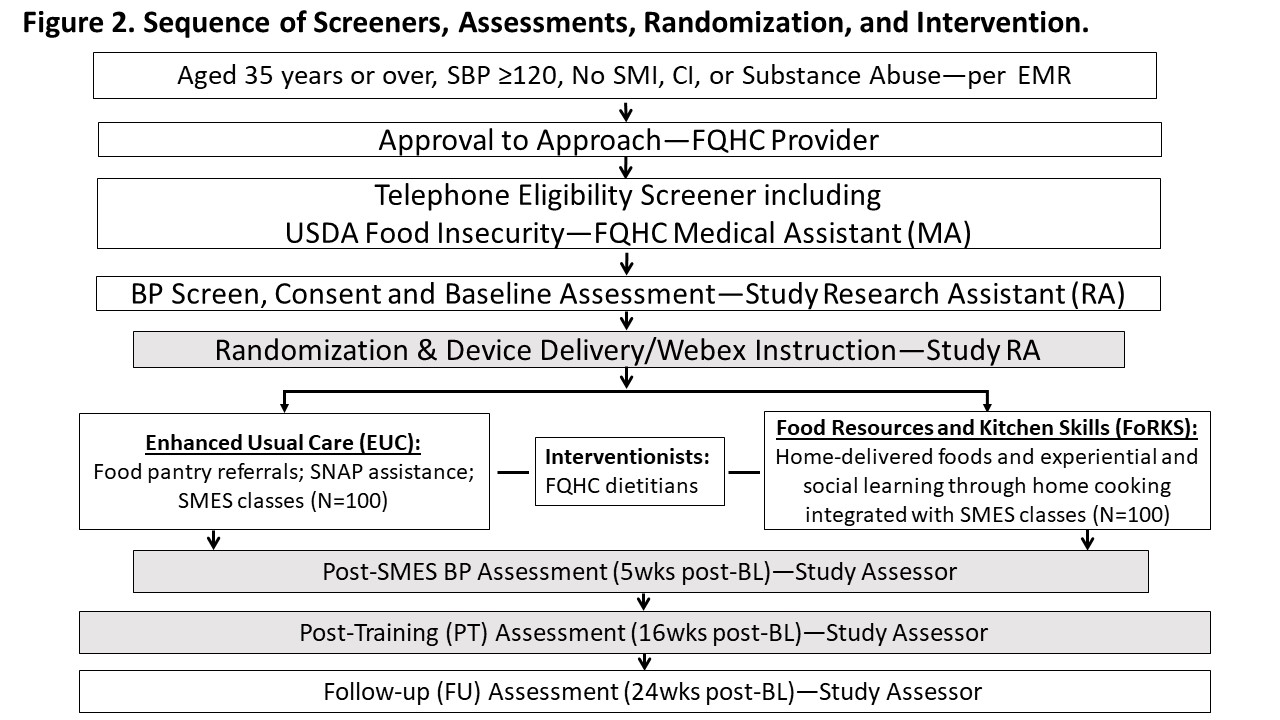


Recruitment will happen in 1 of 4 ways:

1. Potentially eligible adults will be identified from the Eskenazi Epic EMR via Regenstrief Data Services. From this list of patients, Initial study approach and eligibility screening will be made by study personnel.

2. Potentially eligible adults will be identified by FQHC providers who will conduct initial approach with basic study information and provide study flyer. For interested patients, FQHC providers will securely send contact information to study personnel for eligibility screening.

3. Self-referral. Study flyers will be available in Eskenazi health centers and Fresh for You Markets. In addition, the WRTV news feature about Eskenazi’s programming for food insecurity may be run in Eskenazi clinics and Fresh for You Markets to promote the study. Patients may initiate contact with study team for participation.

4. Patients who enrolled under FoRKS+ (IRB#16547) who failed to meet inclusion criteria for 24-hr ambulatory blood pressure measure (ABPM) systolic average will be approached for FoRKS by study personnel during FoRKS+ eligibility results visit or in a follow-up phone call. Remaining FoRKS eligibility criteria will be assessed by study team.

For recruitment methods #1-3, patients passing the telephone screen will be invited to schedule an Eligibility Visit for informed consent and 24-hour ABPM initiation. After 24 hours, patients who meet criteria for ABPM will be asked to complete the Baseline (BL) assessment, including all other outcome measures described in Section 5.

For recruitment method #4, ABPM from FoRKS+ eligibility visit will be used to satisfy FoRKS ABPM inclusion criteria for patients meeting eligibility criteria and consenting to the study.

Treatment assignment will occur prior to intervention for all participants in the replicate; block-4 randomization will be used to ensure equal groups. Target randomization is n=200. Randomization will be stratified by race (i.e., Black/all others), sex (male/female), and DM2 status (i.e., DM2 yes/no) to minimize imbalance in treatment assignments in key subgroups. [Participants who are randomized but are unable to start intervention in their replicate due to changes in health status, schedule availability, etc, will be allowed to participate in the immediately subsequent replicate with the same treatment assignment; ABPM and BL assessment will not be repeated. If participant wants to postpone participation until later replicates, ABPM and BL assessment will be repeated.]

Prior to intervention start, all participants will complete a 1-on-1 consult with an Eskenazi dietician to complete an enrollment appointment for Hypertension group. This consult may be completed at an Eskenazi health center or via phone, and take 30-60 minutes to complete.

Intervention will be conducted in replicates targeting 20 participants (+/- 8 participants) with 10 in each arm (+/-4 participants). A Post-training (PT) assessment will be conducted near Week 16 as the primary endpoint, and Follow-Up (FU) assessment will be conducted around Week 24 for data on maintenance of PT outcomes. Adverse events will be recorded any time they become known to study staff.

1. **Intervention**

**Enhanced Usual Care (EUC) - active control arm:**

Participants randomized to EUC will have access to existing usual primary care services. They will be enrolled in Hypertension Self-Management Education and Support (SMES) class (“Hypertension group”), which is an existing CDC-endorsed program offered at Eskenazi to provide information and skills for managing hypertension (HTN). This course is led by Eskenazi registered dieticians (RDs), with physician (MD), PharmD, and health coach assistance. SMES classes may be conducted in person or via Webex once per week for 5 weeks. Internet-enabled smart phones or tablets will be provided by Eskenazi to participants who need it for accessing virtual SMES classes.

HTN SMES content will include:

- Introduction to HTN; Nutrition labels, sodium identification and reduction
- Medication management; Understanding/monitoring blood pressure
- Physical activity recommendations; Group medical visit and risk reduction
- Nutrition DASH; SMART Goals
- Group medical visit; sharing and topic review

Operations for this arm will be combined with EUC arm from IRB #16547 as the course is led by the same RDs and contains the same content. This arm may also include other Eskenazi patients enrolled in Hypertension group who are not study participants.

BP and pulse will be collected near completion of Week 5 class. In addition, starting in week 6, EUC participants will receive a $10 gift card per week through week 24; when possible, reloadable cards will be used.

**Food Delivery and Cooking (FoRKS) – active intervention arm:**

Participants randomized to FoRKS will receive internet-enabled (via cellular data) tablet devices and tablet stands for video conferencing and remote participation. Training on the device will be completed either in person or via phone.

In Weeks 1-5, participants randomized to FoRKS will attend weekly HTN SMES classes *separately* from EUC participants. SMES classes will include the EUC curriculum stated above and an introduction to the upcoming FoRKS intervention. BP and pulse will be collected near the completion of Week 5 class.

In Weeks 6-16 (“Extended Group Training”), FoRKS continues with home-delivered Mediterranean-style ingredient kits, food management lessons, and hands-on cooking classes in one’s own kitchen. Classes are led by RDs via Webex from physical space within an FQHC facility. Classes are held twice per week thru Week 12, then only once per week thru Week 16; weeks with holidays may have classes cancelled or rescheduled at the discretion of the dietician. Classes are supported by study-provided cooking resources:

- - Participants will receive *fresh*, home delivered and Mediterranean-style ingredient kits weekly thru Week 13, and are responsible for securing their own ingredients in Week 15-16 (no cooking in Week 14).
- Each participant will receive a set of kitchen tools and utensils to participate in cooking classes (e.g. cutting board, chef’s knife, paring knife, spatula, mixing spoon, measuring spoons, liquid and dry measuring cup, can opener, strainer, saucepan, skillet, mixing bowls, zester, vegetable peeler, and meat thermometer).

Classes may include: discussion of the day’s recipe (nutrition, ingredients) and needs for preparation (kitchen equipment, safe/sanitary space), hands-on cooking (instruction and demonstration of tools and various cooking techniques), lessons on budgeting, meal planning, and shopping, and a virtual group meal (sample the meal together and share thoughts about the food and lesson).

Weeks 1-16 operations for this arm will be combined with FoRKS arm from IRB #16773 as the course is led by the same RDs and contains the same content.

Every 2-3 weeks, participants will connect with study personnel and RD’s (via home visits, video conference, or phone calls) throughout the intervention setup and trial to provide feedback on their experience and receive guidance on kitchen workspace prep and organization.

Engagement and satisfaction in these weeks are enhanced through communication games interspersed with milestone activities (e.g., party hats or t-shirts worn in session).

In Weeks 17 through 24 (“Maintenance”), classes and deliveries end, but participants will have continued access to recipes, including ingredient lists and shopping tips. FoRKS participants will also receive a $10 gift card each week of the maintenance period; when possible, reloadable cards will be used.

**Strategies for Treatment and Measurement Fidelity.** FoRKS RD’s will deliver the FoRKS intervention only. No interventionist will act as study assessor and no assessor will act as an interventionist. Assessors will complete a certification process prior to assessing enrolled participants. After certification, ongoing quality assurance checks will continue throughout the study. Assessors will remain masked to study condition.

1. **Measures and Outcomes**

Outcome assessments will be completed in home, at the FQHCs, or by telephone, using Research Electronic Data Capture (REDCap) direct data entry when appropriate.

Assessments occur at Eligibility Visit (EV), BL (week 0), post SMES (target week 5; window Wk 5-6), PT (target week 16; window Wk 15-18), *and/or* FU (target week 24; window Wk 23-26) *as indicated below*. These assessments, excluding the ASA-24, require about one hour.

**Feasibility and Adherence.** We will document number of potentially eligible per Epic EMR, number assessed for eligibility by phone, and reasons for ineligibility or refusal. We define *reach* as the percent of those eligible that enroll, are randomized, and participate in one or more intervention activities.

**Demographics (BL only)**. Sample descriptors and possible covariates including age, sex, race, ethnicity, household income, education, household size, and marital status will be confirmed or self-reported at BL.

**Health (BL, PT, and FU)**. We will assess physical activity via the Stanford Brief questionnaire and usual number of cigarettes/day via the NHANES format to assess intervention carry-over to general health behaviors. Alcohol consumption is included in the ASA-24 and prescription medication changes are captured by the EMR. Body weight will be measured by Tanita WB-800S scale (capacity of 660lbs +/- 0.1lbs).

**Mood and Health-Related Quality of Life.** We will include the PHQ-8 and Generalized Anxiety Disorder measures, as well as the EQ-5D-5L health outcomes questionnaire. Participants scoring ≥ 12 on PHQ-8 or > 15 on GAD-7 will be instructed to contact their PCP and notified that the internal safety officer will also alert the participant’s PCP of their high score.

**Blood Pressure & Pulse (BL, PT, FU, and post SMES).** We will measure BP and pulse using standard procedures with an Omron series 10 device and calculate pulse pressure (i.e., the difference between systolic and diastolic BP). Blood pressure will be measured 3 times at each assessment.

**ABPM (EV and PT).** We will also measure 24-hour blood pressure using the Spacelabs Healthcare ambulatory blood pressure monitor (ABPM), once at Eligibility Visit and once at PT. Appropriately sized cuffs will be used with bladder sizes that encircle 80–100% of arm circumference and widths that are at least 40% of arm circumference. We will measure ambulatory BP three times per hour during participant’s normal daytime hours and two times per hour during participant’s normal nighttime (i.e., sleep) hours. Resting heart rate is recorded by the Spacelabs device.

**HbA_1c_ (BL, PT, FU).** We will measure HbA1c with 1 μL of whole-blood from a fingerstick using a point-of-care analyzer at each assessment. Manufacturer operation guidelines will be followed for obtaining and analyzing samples. HbA1c values will be shared with participants.  Any participant who has an HbA1c value above 6.5% will be given a recommendation to communicate with their PCP.  For any participant with a value above 9%, our internal safety officer will share the value and collection method directly with the PCP as well.

Any participant who had venous whole blood collection prior to this protocol change will have blood collected at each remaining visit in their schedule of events. For these participants, phlebotomists will draw the blood, mix by inversion 4-5 times, then freeze on dry ice for transport to the laboratory. Specimens will be stored at -80°C until the time of assay. HbA_1c_ measures will be performed in the Analyte Lab (Considine Laboratory) at the Center for Diabetes and Metabolic Diseases at Indiana University.

**Social Support, Food-Related Self-Efficacy and Food Resource Management (BL, PT, and FU).** Social support is a multi-dimensional construct that refers to social relationships that promote well-being through emotional, informational, and/or instrumental channels. We will use the Social Support and Eating Habits Survey. This 10-item scale captures friend and family support or sabotage of healthy food habits.

Food-related self-efficacy will be assessed by a 9-item questionnaire capturing basic cooking self-efficacy, meal preparation, and meal planning. This scale has been shown to have good test-retest reliability (range 0.46 to 0.91) and high internal consistency (coefficient alpha 0.84 to 0.86) and to capture change over time in response to cooking lessons.

Food resource management will be assessed using the Plan, Shop, Save, & Cook checklist available on the USDA SNAP-Ed Toolkit site. This 6-item scale includes 5 response options from never to always and contains items such as “how often do you plan meals ahead of time?”, “how often do you compare unit prices before buying food?”, and “how often do you shop with a grocery list?”.

**Food Security, SNAP Usage, Nutrition Literacy, and Diet Quality (BL, PT, and FU).** At all assessments, food security will be assessed with the Four Domain Food Insecurity Scale (4D-FIS). The 4D-FIS items cover shortage of food, unsuitability of food and diet, preoccupation or uncertainty in access to enough food, and lack of control over food situation. The 4D-FIS aligns conceptually with the HFSSM but has more items that yield high factor loadings on the four domains and good internal consistency (Cronbach alphas 0.69 to 0.90).

Health literacy will be tested at BL and PT with the New Vital Sign (NVS). Notably, the NVS involves math word problems that reference an ice cream nutrition label (e.g., if you were allowed 60 grams of carbohydrate as a snack, how much ice cream could you have?).

For diet quality, participants will be asked to complete the ASA-24 (Automated Self-Administered 24-hour Dietary Assessment Tool), National Cancer Institute’s 24-hour dietary recall tool. The ASA24 covers the prior day from midnight to midnight. Accuracy is optimized by the use of images for foods and portion sizes, a large digital library of foods, and the incorporation of the USDA Multiple Pass Method currently used in the NHANES. As has been shown to maximize validity, we will complete 2 interviewer-assisted 24-hour assessments at each time point, which will consist of one weekday and one weekend 24-hour recall when possible. A secure web site allows researchers to register participants, set study parameters, and obtain data files of total kilocalorie and macro and micro nutrient intake.

SNAP knowledge and use will be captured using the semi-structured interview that covers SNAP enrollment, online SNAP use, food outlet type, typical distance and mode of transportation to shop at those stores, and types of food/ingredients purchased with SNAP. For purposes of cost-effectiveness analyses, we will complete a resource use questionnaire and the Short-Form 6D.

**Assessment Incentives.** Participants will receive a $20 gift card for each assessment ($60 total for 3 assessments participation). Participants will receive a $5 gift card for each ASA-24 dietary recall they complete (up to 6 for a total of $30). Gift cards will be given in person or mailed at the completion of the visit.

Participants who do not meet eligibility criteria for the 24-hour ABPM at will be compensated $10 to conclude their participation. Reloadable gift cards will be used when possible.

**Intervention Measures:**

**Engagement** is “the act of being occupied or involved with an external stimulus” and will be assessed in two ways: (1) Participation: Total number of sessions attended; longest streak of consecutive classes attended, per person. This will be recorded for both EUC and FoRKS participants throughout their scheduled intervention. (2) Self-report survey: Total scores on a modified Classroom Engagement Survey, administered approximately every 3 weeks to FoRKS participants during Extended Delivery Training. This structured questionnaire has 16 items that assess, over the prior 3 weeks, the participant’s: affective engagement (e.g., “I feel interested”); behavioral engagement (e.g., “I actively participate in class discussions”); cognitive engagement (e.g., “I go back over things that I don’t understand”); and disengagement (e.g., “I let my mind wander”).

**Participant Experience** will be assessed approximately every 3 weeks to FoRKS participants during Extended Delivery Training and include Likert scale questions regarding satisfaction with foods, class content, instructions, and technology. We will summarize Likert scale scores and use qualitative feedback to make intervention adjustments as the study progresses. Once, at the PT visit, subjects will be asked about any personal “costs” experienced during the FoRKS intervention.

1. **Statistical Considerations**

**Power and Sample Size.** We determined the sample size to ensure adequate power for testing the primary hypothesis on the effectiveness of the proposed FoRKS intervention on systolic blood pressure (SBP). Specifically, we hypothesize that in comparison to EUC, FoRKS participants will have lower SBP (-6.0 mm Hg) at PT. That a difference of 6 mm Hg is clinically meaningful is supported by a 2021 Lancet meta-analysis involving 48 randomized trials and 344,716 participants that showed a reduction of >5 mm Hg in SBP was associated with a 10% reduction in major CVD events regardless of baseline SBP.

To ensure we have adequate power to detect an effect on SBP, we plan to enroll 100 subjects for each treatment group (n=80 per group at PT assuming 20% attrition). Using a conservative estimate of the SD of 12 mm Hg, we estimate that 100 subjects per group (80 after attrition) will give us 88% power to detect a 6 mm Hg difference in SBP between the treatment groups, with a two-sided t-test at 0.05 significance level.

We have not determined the target sample size based on the analytical power for the secondary outcome (HbA_1c_), because we cannot control how many subjects in our sample will have both HTN and DM2.

**Data management.** Assessment data will be directly entered into REDCap, a web-based secure relational data management system for research data. A data dictionary including each interview question and definition for each response will be generated and used to build the REDCap database. The database will include internal error checking algorithms to prevent erroneous data entry or missing data. EMR and biomarker data will be merged with other study data and stored as SAS data sets on secure servers with access limited to data analysts working on this study and those meeting requirements for data sharing (see Data Storage and Sharing document). Video data will be stored using IU’s enterprise instance of OneDrive, in a HIPAA compliant secure folder created by IU for sensitive data.

**Analytic Plan**. The primary analyses will be carried out following an intention-to-treat (ITT) principle. Per-protocol analyses will be performed secondarily as supplements to the ITT analyses.

**Primary Aim.** We will use the trial data to determine the effects of FoRKS intervention on SBP and, secondarily, glucose management. We will test the hypothesis that compared to EUC, FoRKS participants have lower mean SBP at PT. Among those with both HTN and DM2, we will secondarily examine FoRKS’ effect in lowering HbA_1c_. Mean SBP at PT will be compared between the two treatment groups using a t-test. Mean HbA_1c_ will be compared using the same test. A similar analysis will be performed at FU to determine the sustainability of intervention effects on both outcomes. The planned t-tests at the end of the intervention (PT) are to evaluate the effects of the full dose of the intervention and the sustainability of the intervention at FU. This said, in addition to the main analysis described above, we will use mixed-effect models to assess the time course of the intervention effects by modeling repeatedly measured BP (BL, 5wk, PT, and FU) and HbA_1c_ (BL, PT, FU); random subject effects will be included to account for the potential correlation among repeated measures contributed by the same subjects. These secondary analyses will allow us to control the effects of any participant characteristics that are not balanced.

**Intervention Processes**. We will examine behavior change levers that are associated with food security, nutrition, and SBP. While parts of the analyses are exploratory, we are interested in testing hypotheses (H2.1 and H2.2) that FoRKS participants will report greater food-related social support and self-efficacy and food management skills at PT, and these gains will partially account for better food security, nutrition, and SBP in FoRKS participants relative to EUC. To analyze, we will first perform direct comparisons by using t-tests, but also regression analysis using mixed effect models. Specifically, we will enter repeatedly measured food security, nutrition, and SBP as dependent variables in the mixed effect models, and test the effects of food-related social support and self-efficacy, and food management skills. Similarly, to evaluate the hypothesis (H2.3) that FoRKS participants showing greater learning engagement also experience greater social support, self-efficacy, and food management skill gains, we will use regression models to examine the association between learning engagement and social support, self-efficacy, and skill gains in FoRKS participants. Finally, we will explore whether SNAP participation modified intervention effects on food security, nutrition, or SBP.

**Maintenance.** From PT to FU, we will determine and report the percent of FoRKS vs EUC participants who maintain improved food security, nutrition, SBP, and/or HbA_1c_. We will dichotomize changes in these outcomes (maintained/improved outcome vs worsened outcome) between PT and FU. We will report the percentages of maintenance and improvement in each of the outcomes. We will also conduct logistic regression analyses to model the dichotomized outcomes and identify factors associated with improvement in each of the outcomes.

**Cost-Effectiveness.** Finally, in this NIH Stage III behavioral trial, we will provide an initial indication of the cost-effectiveness of FoRKS intervention. We regard this as initial because the sample size is unlikely to provide adequate power for the cost effectiveness analysis (CEA) and we do not expect significant differences in health care use over just 24 weeks. The CEA shall adhere to the best practices of the Consolidated Health Economic Evaluation Reporting Standards.
